# Longitudinal Molecular Epidemiology and Genomic Surveillance of Carbapenem‐Resistant *Klebsiella pneumoniae* in the Gut of Healthy Individuals in China

**DOI:** 10.1002/mco2.70814

**Published:** 2026-08-05

**Authors:** Zelin Yan, Yan Li, Yi Sun, Qiaoling Sun, Congcong Liu, Yanyan Zhang, Yuchen Wu, Hongwei Zhou, Shangshang Qin, Gongxiang Chen, Ruichao Li, Rong Zhang

**Affiliations:** ^1^ Department of Clinical Laboratory, School of Medicine Second Affiliated Hospital of Zhejiang University Hangzhou China; ^2^ School of Pharmaceutical Sciences Zhengzhou University Zhengzhou China; ^3^ Jiangsu Co‐Innovation Center For Prevention and Control of Important Animal Infectious Diseases and Zoonoses, College of Veterinary Medicine Yangzhou University Yangzhou China; ^4^ Department of Laboratory Medicine, the First Affiliated Hospital Zhejiang University School of Medicine Hangzhou China; ^5^ Department of Clinical Laboratory, School of Medicine, Tianjin First Central Hospital Nankai University Tianjin China

**Keywords:** Carbapenem‐resistant *Klebsiella pneumoniae*, Healthy individuals, Carbapenemase genes, ST11, IncHI2‐IncHI2A

## Abstract

Carbapenem‐resistant *Klebsiella pneumoniae* (CRKP) was identified by the World Health Organization in 2024 as a key priority bacterium for the development of novel control strategies. While most surveillance studies have focused on clinical isolates, the prevalence and genomic characteristics of CRKP in healthy populations remain poorly understood. Here, we present a nationwide longitudinal descriptive genomic analysis of CRKP isolates from healthy individuals in China in 2016 and from 2019 to 2024. Within this hospital‐based health‐check cohort, CRKP prevalence was higher in southern regions. Descriptive temporal changes in carbapenemase gene composition were observed, with *bla*_NDM_ variants, especially *bla*_NDM‐5_, more frequently detected in later years and often associated with IncHI2‐IncHI2A plasmids. ST11, primarily represented by capsular types KL47 and KL64, remained the predominant lineage throughout the study period. Gene presence/absence association analysis identified several ST11‐enriched loci related to iron acquisition, capsular polysaccharide synthesis, and secretion‐associated functions, highlighting candidate lineage‐associated features. Comparative genomic analysis with 113 clinical CRKP isolates revealed high genetic similarity (SNP < 100) between some healthy‐carrier and clinical strains, suggesting possible clonal relatedness. These findings indicate that healthy individuals in this hospital‐based cohort can harbor clinically relevant CRKP, supporting surveillance beyond symptomatic infections while emphasizing the descriptive scope of the present study.

## Graphical abstract

Using 16,040 stool samples from healthy individuals across 30 Chinese provinces (2016–2024), we performed longitudinal genomic surveillance of carbapenem‑resistant *Klebsiella pneumoniae* (CRKP) colonizing the gut. We reveal rising community CRKP prevalence, especially in southern China, a shift from *bla*_KPC‐2_ to plasmid‑borne *bla*_NDM‐5_ on IncHI2/IncHI2A backbones, and the persistence of ST11 (KL47/KL64) lineages enriched for colonization‐associated genes, highlighting the healthy gut as a reservoir for clinically relevant CRKP.

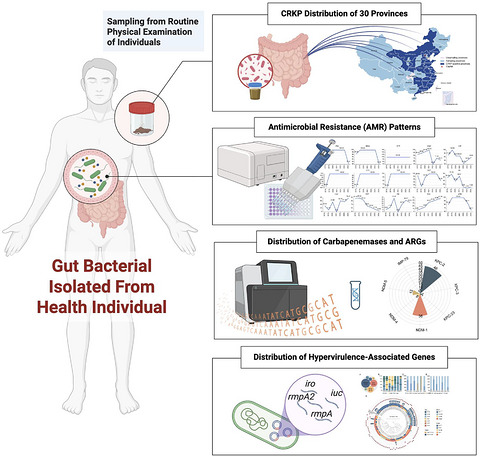

## 1 Introduction

*Klebsiella pneumoniae* is an opportunistic pathogen capable of causing severe infections such as respiratory tract, bloodstream, and urinary tract infections [1]. This species has gained notoriety for its multidrug resistance, hypervirulence, and high transmissibility, including both intra‐ and interspecies dissemination, posing a substantial threat to public health [2]. Due to limited therapeutic options, infections caused by these strains are often difficult or even impossible to treat in hospitalized patients. Carbapenems are regarded as the last line of defense against multidrug‐resistant *K. pneumoniae*; however, the widespread and inappropriate use of these antibiotics in clinical settings has led to a significant rise in carbapenem‐resistant *K. pneumoniae* (CRKP), markedly increasing treatment failure and mortality rates [3].

In the chronicles of public health, the rise and swift dissemination of CRKP within hospital settings have posed a formidable challenge [4]. This is particularly alarming due to the paucity of effective antibacterial treatments, which has led to the identification of an increasing number of CRKP strains characterized by multidrug resistance and hypervirulence [5]. The 2024 CHINET report underscores a stark reality: *K. pneumoniae* has risen to the second most prevalent bacterium in Chinese clinics, with a significant escalation in resistance to meropenem and imipenem, jumping from 2.9% and 3.0% in 2005 to alarming levels of 20.5% and 21.2% in 2024 (http://www.chinets.com). The rising levels of antibiotic resistance in CRKP severely restrict available therapeutic options and significantly increase the risk of treatment failure, underscoring the urgent need for continuous surveillance. Ongoing monitoring of CRKP is essential to inform clinical decision‐making and guide the rational selection of effective antimicrobial therapies.

CRKP is widely distributed across diverse hosts, with clinical patients representing a major reservoir [6]. CRKP can trigger nosocomial outbreaks through direct transmission or environmental contamination, and hospital‐derived strains may further disseminate into aquatic systems via wastewater discharge. Although the use of carbapenems is strictly prohibited in livestock production, CRKP has been reported in various animals and animal‐derived products. Notably, carbapenem resistance genes in animals can be transferred to humans via mobile genetic elements such as plasmids, accelerating the spread of resistance.

While hospital settings remain the primary focus of CRKP surveillance, increasing evidence suggests that healthy individuals can carry multidrug‐resistant Enterobacterales and may contribute to their circulation across healthcare‐associated and community‐facing settings [7]. Asymptomatic carriage in the gut provides an ecological niche for the maintenance and exchange of resistance genes and mobile genetic elements [8]. However, the epidemiological relationship between colonizing strains in healthy individuals and clinically derived CRKP remains insufficiently defined at the national level. Therefore, understanding the prevalence, genomic characteristics, and longitudinal patterns of CRKP in healthy populations is important for broadening surveillance beyond symptomatic infections [9].

Although multiple studies have systematically explored CRKP epidemiology in clinical and animal populations, comprehensive nationwide analyses specifically addressing CRKP colonization among healthy human populations are extremely limited [10, 11]. To address this knowledge gap, we conducted a nationwide, retrospective epidemiological survey of healthy individuals from 30 regions in China between 2016 and 2024. By integrating molecular and genomic approaches, we characterized the epidemiological and genomic features of CRKP isolates from the healthy population. Additionally, comparative genomic analysis was used to identify genomic features associated with the ST11 background in healthy‐carrier isolates. These findings provide essential insights for developing evidence‐based strategies to prevent and control CRKP transmission and infection.

## 2 Results

### 2.1 Nationwide Trends and Regional Variations of CRKP Distribution

**FIGURE 1:**
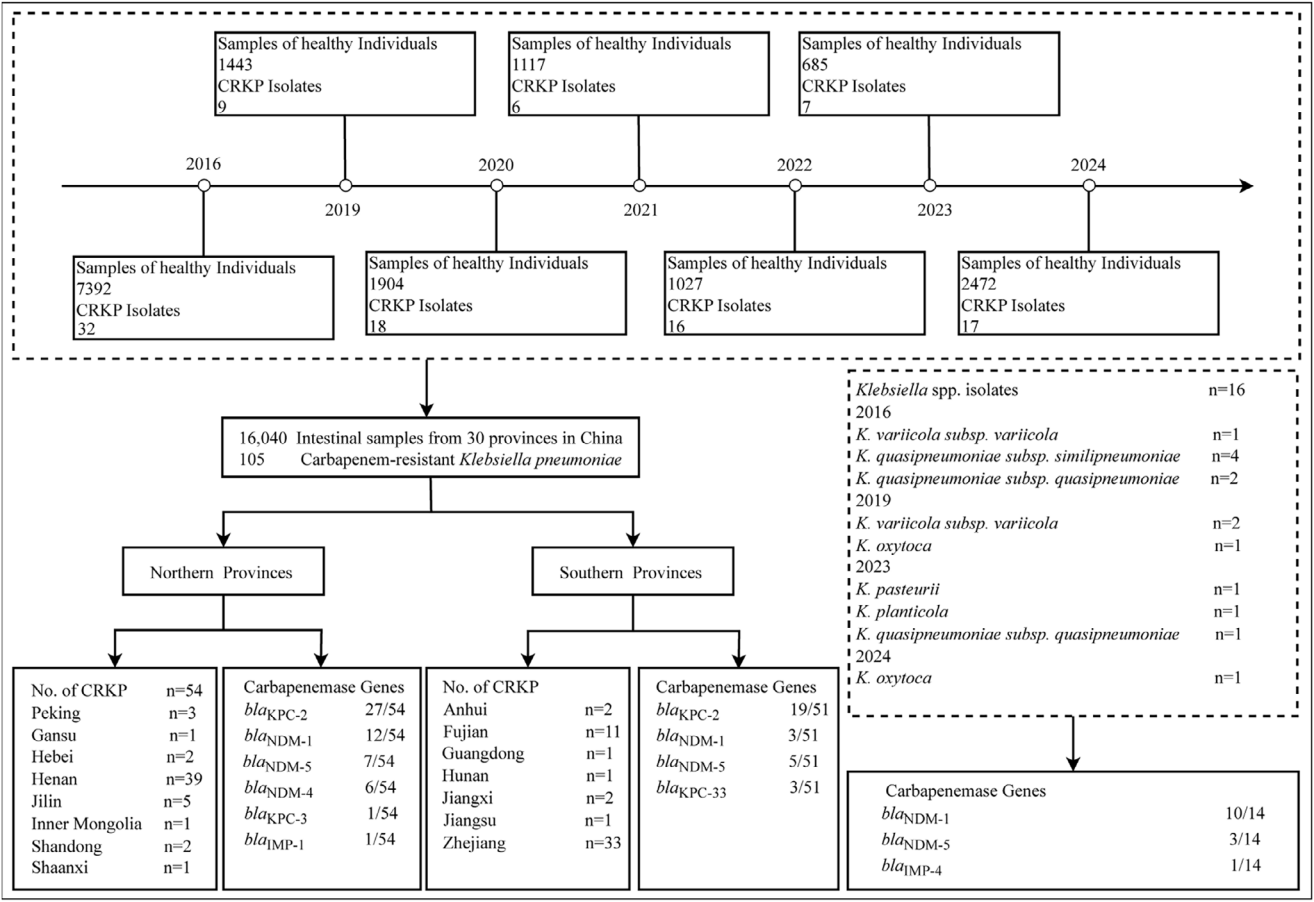
Timeline and geographical distribution of CRKP isolated from healthy individuals in China (2016, 2019 to 2024).

**FIGURE 2:**
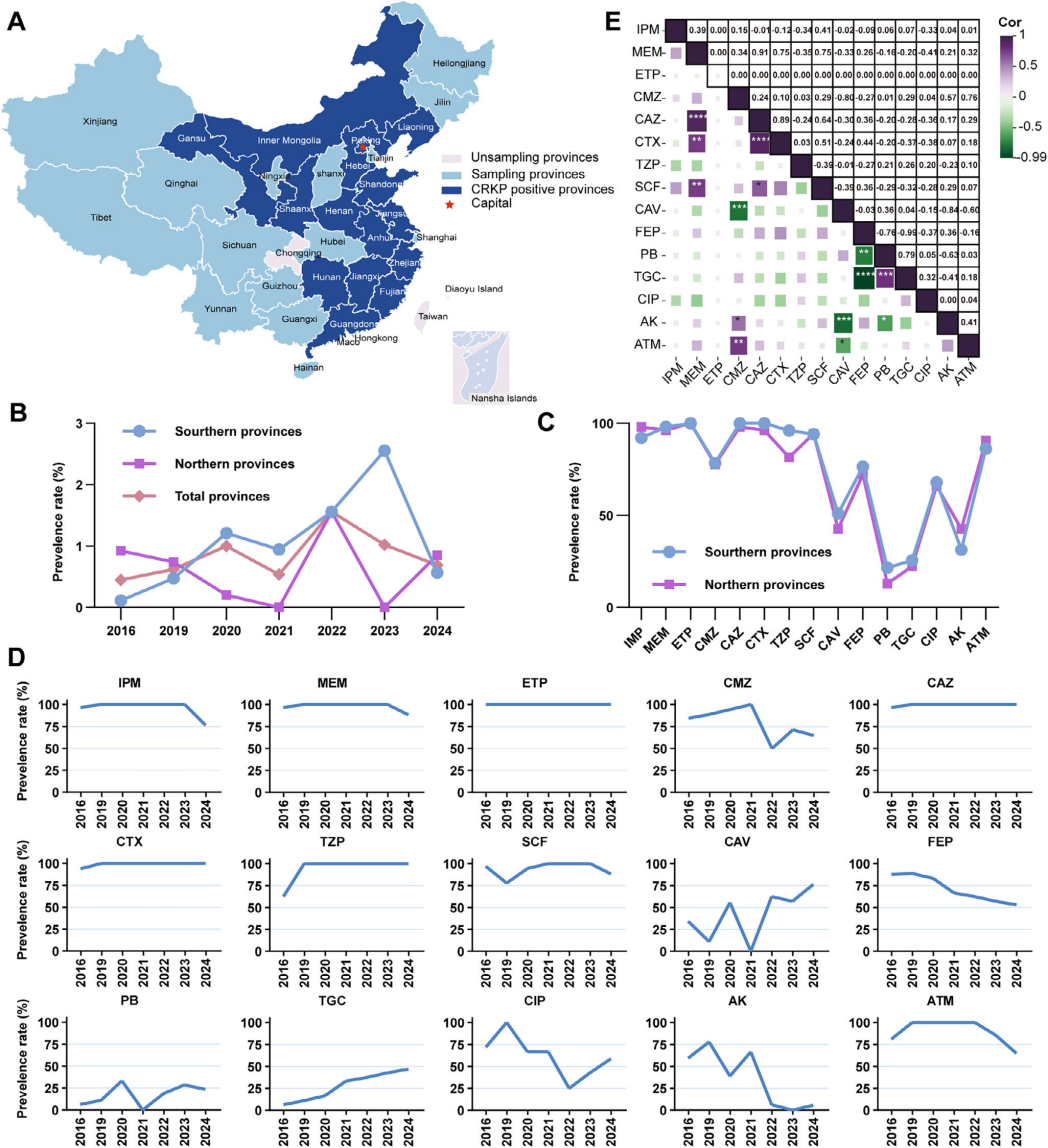
Epidemiological and resistance trends of CRKP in China over eight years, and correlations between 15 antimicrobial resistance rates. (A) The map shows the spatial distribution of CRKP isolates across northern and southern provinces (China Map Approval Number: GS(2019)1682). (B) A line chart depicting CRKP isolation rates from 2016, 2019 to 2024, stratified by northern and southern regions. (C) A line chart comparing the temporal trends of resistance rates for 15 types of key antibiotics between northern and southern regions. The figure illustrates regional differences in antimicrobial resistance patterns across the study period. (D) Temporal trends in resistance rates for key antibiotics in all isolates are illustrated. (E) A heatmap depicting the correlation matrix among resistance rates for different antibiotics.

During 2016 and 2019–2024, we isolated 105 CRKP isolates from a total of 16,040 intestinal samples across 30 provinces in China, yielding an overall prevalence of 0.65% (Figure 1; Table 1; Table S1). Additionally, 16 isolates of other *Klebsiella* subspecies were detected, showing a declining trend over time. CRKP prevalence fluctuated over time, peaking at 1.56% in 2022, followed by a slight decline. Within this hospital‐based cohort, CRKP prevalence was generally higher in southern regions, reaching its highest observed levels in 2022 (1.56%) and 2023 (2.55%) (Figure 2A,B). However, this year‐to‐year regional pattern should be interpreted cautiously, as changes in the composition of contributing provinces and hospitals over time may have influenced the observed trend.

**TABLE 1:** Summary characteristics of 105 CRKP isolates.

Variable	Category	Number of isolates (%)	Dominant STs (number)	Dominant carbapenemase genes (number)
Geographic group	North China	54 (51.4)	ST11 (28); ST252 (6); ST1 (4)	KPC‐2 (27); NDM‐1 (12); NDM‐5 (7); NDM‐4 (6)
	South China	51 (48.6)	ST11 (20); ST2270 (6); ST179 (4)	NDM‐1 (24); KPC‐2 (19); NDM‐5 (5); KPC‐33 (3)
Province (top five by isolate number)	Henan	39 (37.1)	ST11 (23); ST252 (6)	KPC‐2 (23); NDM‐1 (7); NDM‐4 (6)
	Zhejiang	33 (31.4)	ST11 (10); ST2270 (6)	NDM‐1 (18); KPC‐2 (11); NDM‐5 (3)
	Fujian	11 (10.5)	ST11 (5); ST179 (4)	NDM‐1 (6); KPC‐2 (3); KPC‐33 (2)
	Jilin	5 (4.8)	ST1 (3); ST1449‐1LV (2)	NDM‐1 (3); NDM‐5 (2)
	Beijing	3 (2.9)	ST45 (1); ST1 (1)	NDM‐5 (1); NDM‐1 (1); KPC‐3 (1)
Carbapenemase gene	KPC‐2	46 (43.8)	ST11 (45); ST15 (1)	North China (27); South China (19)
	NDM‐1	36 (34.3)	ST2270 (6); ST1 (4); ST37 (4)	South China (24); North China (12)
	NDM‐5	12 (11.4)	ST782 (2); ST1449‐1LV (2); ST45 (1)	North China (7); South China (5)
	NDM‐4	6 (5.7)	ST252 (6)	North China (6)
	KPC‐33	3 (2.9)	ST11 (2); ST15 (1)	South China (3)
	KPC‐3	1 (1.0)	ST11 (1)	North China (1)
	IMP‐79	1 (1.0)	ST70‐1LV (1)	North China (1)
Sequence type (top ten)	ST11	48 (45.7)	−	KPC‐2 (45); KPC‐33 (2); KPC‐3 (1)
	ST252	6 (5.7)	−	NDM‐4 (6)
	ST2270	6 (5.7)	−	NDM‐1 (6)
	ST1	4 (3.8)	−	NDM‐1 (4)
	ST15	4 (3.8)	−	NDM‐1 (1); KPC‐2 (1); KPC‐33 (1)
	ST37	4 (3.8)	−	NDM‐1 (4)
	ST179	4 (3.8)	−	NDM‐1 (4)
	ST34	2 (1.9)	−	NDM‐1 (2)
	ST45	2 (1.9)	−	NDM‐5 (1); NDM‐1 (1)
	ST782	2 (1.9)	−	NDM‐5 (2)
Antimicrobial susceptibility	IPM	R 100 (95.2)	−	−
	MEM	R 102 (97.1)	−	−
	ETP	R 105 (100.0)	−	−
	CAZ	R 104 (99.0)	−	−
	CTX	R 103 (98.1)	−	−
	TZP	R 93 (88.6)	−	−
	FEP	R 78 (74.3)	−	−
	PB	R 18 (17.1)	−	−
	TGC	R 25 (23.8)	−	−
	CIP	R 65 (61.9)	−	−
	AK	R 39 (37.1)	−	−
	ATM	R 92 (87.6)	−	−

*Note*: More details in Table S1.

Abbreviations: AK, amikacin; ATM, aztreonam; CAV, ceftazidime/avibactam; CAZ, ceftazidime; CIP, ciprofloxacin; CMZ, cefmetazole; CRKP, carbapenem‐resistant *Klebsiella pneumoniae*; CTX, cefotaxime; ETP, ertapenem; FEP, cefepime; IPM, imipenem; MEM, meropenem; PB, polymyxin B; R, resistant; I, intermediate; S, susceptible; SCF, cefoperazone/sulbactam; ST, sequence type; TGC, tigecycline; TZP, piperacillin/tazobactam.

### 2.2 Temporal Trends and Regional Characteristics of Antibiotic Resistance

The 105 CRKP isolates exhibited significant temporal and regional variations in antimicrobial resistance (AMR) patterns, consistently showing high resistance (>90%) to carbapenems (IPM, MEM, ETP) and cephalosporins (CTX, CAZ) (Table 1; Table S1; Figure 2C). Temporal analyses highlighted a continuous and significant increase in tigecycline (TGC) resistance (Tau = 1.000, *p* = 0.0004), whereas resistance to cefepime (FEP) significantly declined (Tau = −0.905, *p* = 0.0028) (Figure 2D). Southern isolates demonstrated higher resistance rates to multiple antibiotics, especially cefoperazone‐sulbactam (SCF) and ciprofloxacin (CIP), compared with northern isolates. However, a similar increasing trend in TGC resistance was noted in both regions. Correlation analysis indicated strong associations between carbapenem antibiotics and a possible trade‐off between TGC and FEP resistance mechanisms, possibly due to selective pressure dynamics (Figure 2E).

### 2.3 Carbapenemase Gene Dynamics and Plasmid‐Mediated Dissemination

**FIGURE 3:**
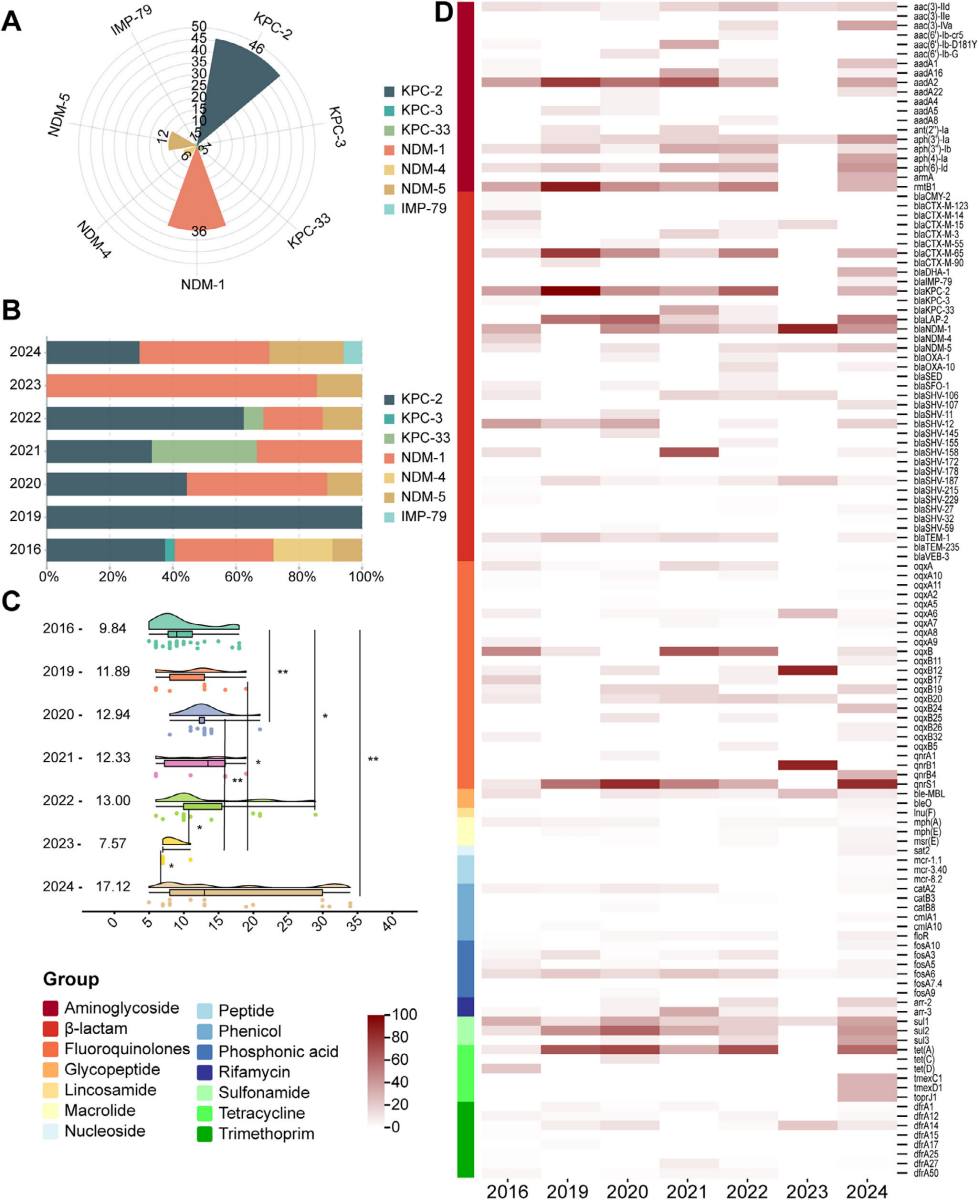
Distribution of carbapenemases and ARGs in CRKP isolates from healthy individuals in China. (A) Rose diagram of carbapenemase distribution. (B) Bar graph displaying the percentage of different carbapenemases by year. The *y*‐axis represents the year of isolation, and the *x*‐axis represents the percentage of carbapenemases. Different colors represent different carbapenemases. (C) The distribution of the number of ARGs per isolate in different time groups. (D) ARGs prevalence grouped by sampling period. ARGs clustered by antimicrobial resistance groups and colored by antimicrobial classes. The percentage indicates the proportion of isolates within each sampling period that carried the corresponding ARG.

Seven carbapenemase genes were identified among 105 CRKP isolates from healthy individuals, with *bla*_KPC‐2_ being most prevalent (43.81%), followed by *bla*_NDM‐1_ (34.29%) and *bla*_NDM‐5_ (11.43%) (Figure 3A). Among the carbapenemase genes detected across different years, *bla*_KPC‐2_ and *bla*_NDM‐1_ consistently dominated (Figure 3B). Distinct geographical patterns emerged, with *bla*_KPC‐2_ (27/54, 50.0%) predominant in northern regions and *bla*_NDM‐1_ (24/51, 47.0%) in the south. Over the study period, descriptive temporal changes in carbapenemase gene composition were observed, including an increased representation of *bla*_NDM_ variants, particularly *bla*_NDM‐5_, in later years (Figure 3D; Table 1; Table S1). *bla*_NDM‐5_ was frequently associated with IncHI2‐IncHI2A plasmids (*R* > 0.8, *p* < 0.05), suggesting a potential contribution of these plasmids to dissemination in this cohort.

To further validate the relationship between *bla*_NDM‐5_ and IncHI2‐IncHI2A plasmids, we conducted third‐generation Nanopore sequencing on the *bla*_NDM‐5_‐positive strain H22‐ZJ03, which revealed that *bla*_NDM‐5_ was located on a 268‐kb IncHI2‐IncHI2A plasmid pH22‐ZJ03‐NDM‐5 (Figure S1). Moreover, 58.33% of *bla*_NDM‐5_‐positive isolates carried IncHI2‐IncHI2A plasmid replicons. The circular map results showed that, with the exception of NJR‐C‐106, the sequences of other isolates exhibited high similarity to plasmid pH22‐ZJ03‐NDM‐5. Additionally, conjugation experiments demonstrated successful transfer of *bla*_NDM‐5_ plasmids to the recipient strain YZ6, whereas no transconjugants were obtained for *bla*_IMP_ and *bla*_KPC_ positive isolates under the experimental conditions used. Transconjugants exhibited resistance profiles similar to donor strains, including resistance to ceftazidime/avibactam and meropenem (Table S2).

### 2.4 Dynamics of Antibiotic Resistance Genes, Virulence Factors, and Capsular Types

The total number of ARGs in CRKP isolates increased significantly over the study period, with a significant rise observed in 2024 compared with 2016 and 2023 (*p* < 0.05) (Figure 3C). Resistance genes such as *floR*, *aph(3')‐Ia*, and *bla*_NDM‐5_ steadily rose over time, with colistin resistance gene *mcr* and tigecycline resistance gene *tmexCD‐toprJ* newly identified in 2024 (Figure 3D). In contrast, the number of virulence genes fluctuated without a clear trend, although notably higher counts were observed in isolates from 2019 and 2022 compared with earlier years (*p* < 0.05) (Figure S2). Recent years have shown a declining trend in the number of virulence genes carried by isolates.

**FIGURE 4:**
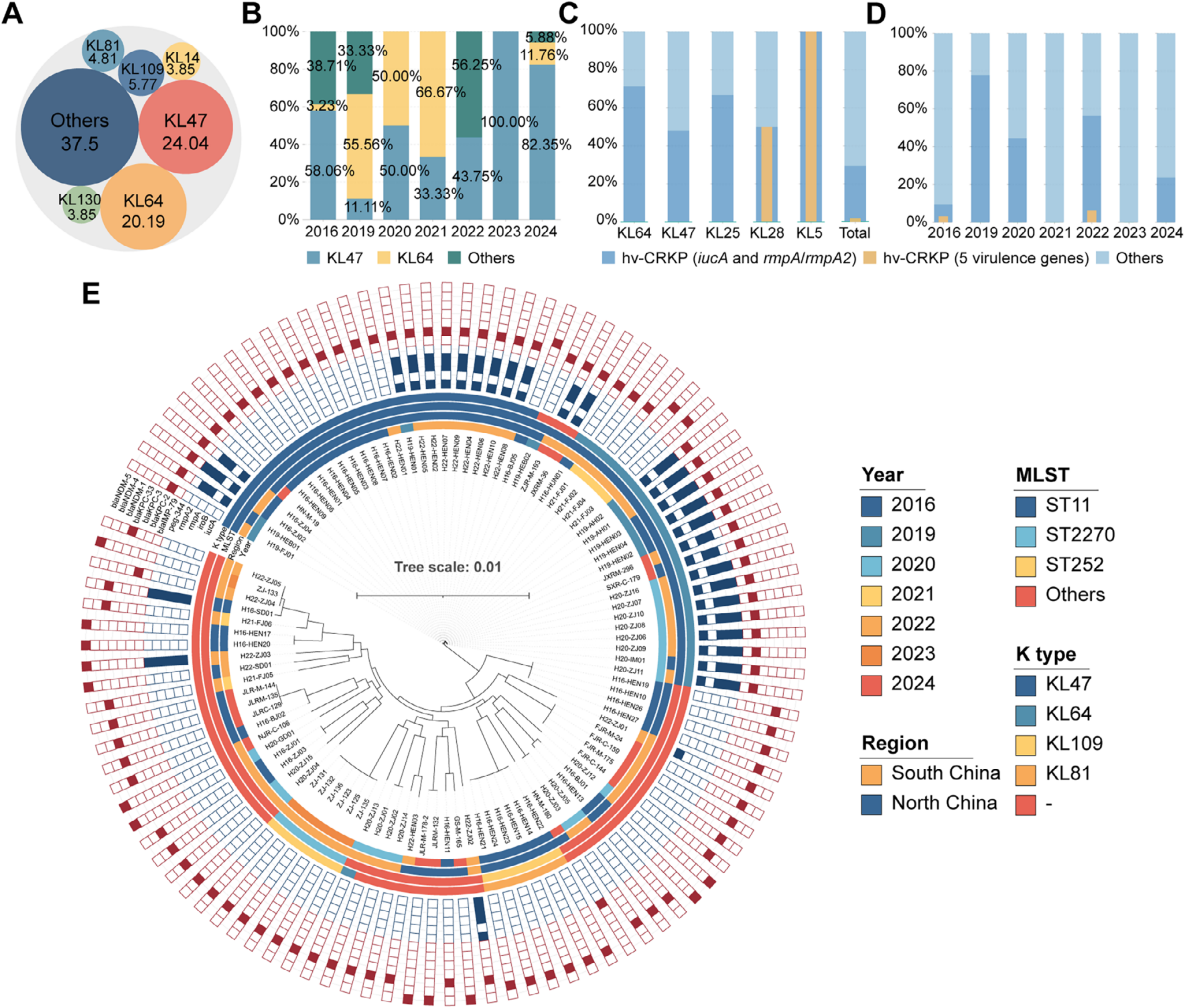
Distribution of hypervirulence‐associated genes among CRKP strains. (A) Distribution of K types in CRKP isolates. (B) Percentage bar chart showing the distribution of different K types across various years, with different colors representing distinct K types. (C) Proportion of hv‐CRKP among CRKP isolates in China across different years, hv‐CRKP was defined as hvKP isolates carrying carbapenem‐resistance genes. (D) Proportion of hv‐CRKP within different K‐type CRKP isolates in China. (E) Phylogenetic analysis of 105 CRKP isolates: Gene presence and absence are indicated by filled and open squares, respectively. In the K type group, '‐' represents other K types.

Multiple K types were identified among CRKP isolates, predominantly KL64 and KL47 (Figure 4A,B). Since 2022, KL47 has emerged as the dominant type among healthy individuals. The hv‐CRKP accounted for 29.52% of isolates, with KL64 (15/31, 48.39%) and KL47 (12/31, 38.71%) as the main K types (Figure 4C,D). Yearly analysis revealed that the proportion of hv‐CRKP was highest in 2019, followed by a decreasing trend over time. Geographical differences in capsular types were also observed, with KL47 (40·74%) dominant in the north and KL64 (31.37%) prevalent in the south. In addition, compared with the 15 capsular types identified in CRKPs of North China, a total of 19 capsular types were detected in CRKPs of South China, with four capsular types shared between the two regions (Figure S3).

**FIGURE 5:**
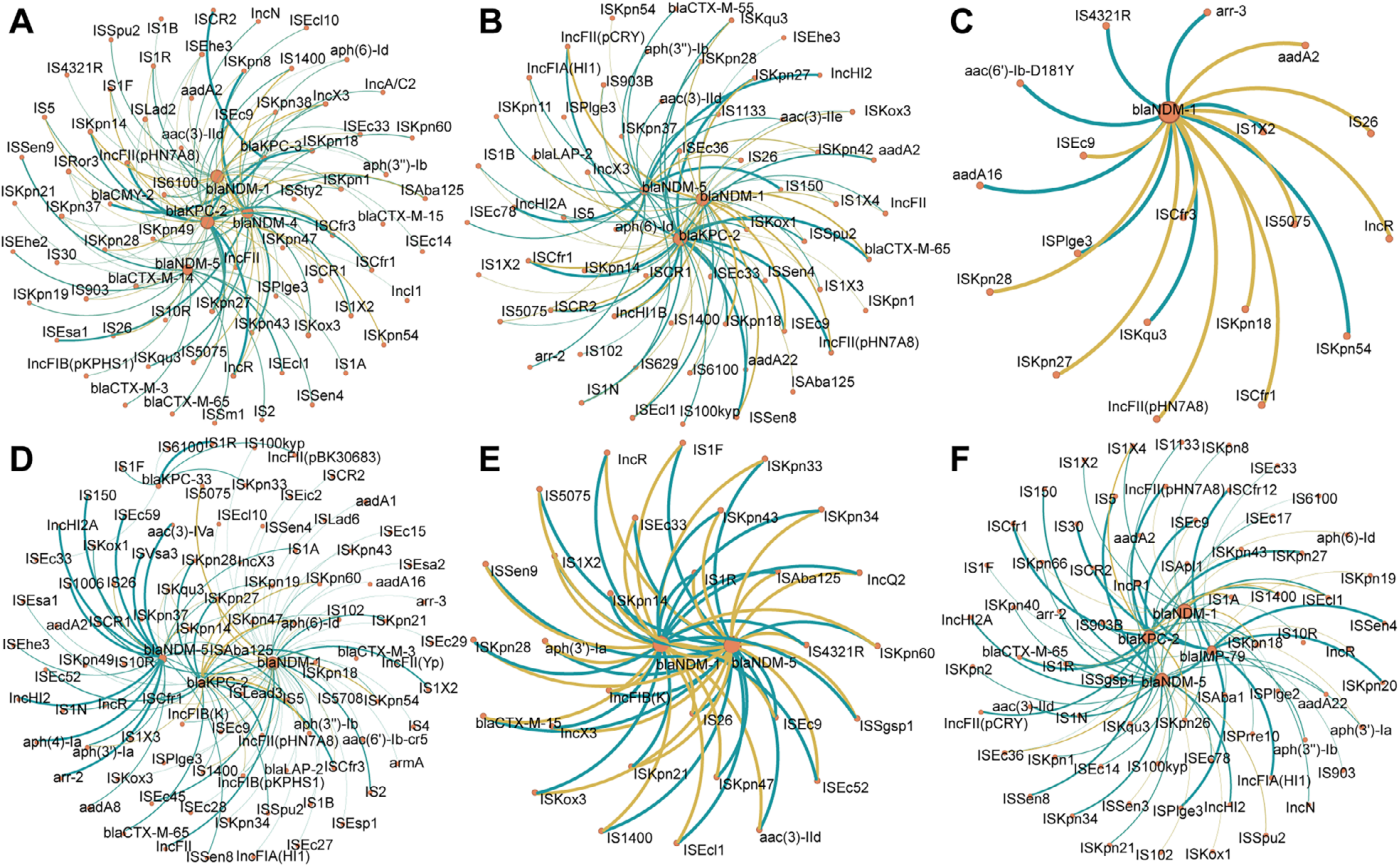
The network graph describing the co‐occurrence pattern of carbapenemase genes with ARGs, ISs, and plasmid replicons. (A–F) The correlation of seven carbapenemase genes with other ARGs, insertion sequences, and plasmid replicons in different years (2016, 2020–2024). No mobile elements significantly associated with the carbapenemase gene were counted in 2019. The nodes represent ARGs, plasmid replicons, or insertion sequences. Connections between nodes indicate that they are related. Green colors and thicker lines represent stronger positive correlations. The yellow color depth on the line shows the strength of the negative correlation; darker yellow indicates a stronger correlation. Moreover, the line's thickness is proportional to the correlation strength, with a thicker line indicating a stronger relationship between the variables. All associated genes in the figure had *p*‐values less than 0.05.

Correlation analyses revealed a significant association between ARG numbers and both plasmid replicons (rSpearman = 0.34, *p* < 0.001) and insertion sequences (rSpearman = 0.39, *p* < 0.001), suggesting their roles in ARG dissemination (Figure S4). However, no significant correlation between ARGs and virulence factors was observed (rSpearman = 0.06, *p* > 0.05). Network analyses further indicated specific correlations between major carbapenem resistance genes (*bla*_KPC‐2_, *bla*_NDM‐5_, and *bla*_NDM‐1_) and various ARGs, insertion sequences, and plasmid replicons, underscoring the complex genetic interactions driving CRKP resistance evolution (Figure 5). For example, *bla*_NDM‐5_ exhibited strong positive correlations with several aminoglycoside resistance genes (*aac(3)‐IVa*, *aph(4)‐Ia*, *aph(6)‐Id*, etc.) and ISs (IS*150*, IS*Vsa3*, and IS*26*, etc.), as well as with IncHI2A and IncHI2 plasmids across multiple years (R > 0.8, *p* < 0.05).

### 2.5 Clonal Shift and Long‐Term Colonization Characteristics of ST11

Phylogenetic analysis based on core genome SNPs demonstrated substantial genomic diversity among the 105 CRKP isolates, identifying 26 known STs (Figure 4E). ST11 was the predominant type (45.7%), mainly associated with capsular types KL47 and KL64. Notably, KL47 progressively replaced KL64 as the dominant capsular type. Genetic similarity (SNPs < 100) across isolates from different years and regions suggested that closely related ST11 CRKP were detected repeatedly in the healthy‐carrier population over time (Table S3).

Given the dominance of ST11 (45.71%, 48/105), we further characterized its genetic features, revealing a fluctuating trend of ARGs number over time with a significant increase in 2024 compared with 2016 (*p* < 0.05) (Figure S6). Correlation analysis revealed a positive association between ARGs and insertion sequences as well as plasmids (Figure S7). In terms of virulence genes, isolates from 2020 harbored the highest number of virulence genes, significantly exceeding those from 2021, 2022, and 2024 (*p* < 0.05).

Compared with non‐ST11 isolates, ST11 CRKP showed enrichment of a large number of loci in the gene presence/absence association analysis. A total of 1229 genes differed significantly between ST11 CRKP and the non‐ST11 group (Bonferroni test, *p* < 0.01). The results revealed that several key genes influencing *K. pneumoniae* colonization were significantly enriched in the ST11 CRKP group compared with the non‐ST11 group (Table S4). These genes were associated with functions such as capsular polysaccharide biosynthesis (*wcaJ*), iron acquisition (*fyuA/psn*, *ybtETUAQXSP*, *irp1*, *irp2*, *iucABCD*, and *iutA*), and the type VI secretion system (*vipB/tssC*, *clpV/tssH*, *icmF/tssM*, and *impA/tssA*). These findings highlight candidate ST11‐associated genomic features; however, because the analysis did not explicitly control for population structure, lineage confounding cannot be excluded.

### 2.6 Genomic Comparisons Between Healthy and Clinical ST11 CRKP Isolates

To further compare the genomic differences between ST11 CRKPs from healthy individuals and clinical patients, we randomly selected 113 CRKP genome sequences with clinical patient source information from the NCBI database (Table S5). These isolates were predominantly from China, followed by the United Kingdom. Phylogenetic tree analysis revealed that CRKPs from the same source did not cluster independently (Figure S7), with some CRKPs from healthy individuals showing high genetic similarity to those from clinical patients (SNPs < 100), suggesting possible clonal relatedness rather than providing evidence for transmission directionality (Table S6). CRKPs from healthy individuals carried significantly fewer ARGs compared with clinical isolates (*p* < 0.05). However, no significant differences were observed in the number of virulence genes between the two groups (*p* > 0.05). In addition, we analyzed the differences in the presence of five key virulence genes in ST11 CRKP isolates from patients and healthy individuals. Statistical analysis using the chi‐squared test showed that only the *iroB* gene was significantly more common in isolates from clinical patients (*χ*2 test, *p* < 0.05), while the other four virulence genes showed no statistically significant difference between the two groups (*p* > 0.05).

## 3 Discussion

This nationwide surveillance study systematically characterized the epidemiological distribution, antimicrobial resistance profiles, and genomic characteristics of CRKP isolates collected from healthy individuals across 30 provinces in China from 2016, 2019 to 2024. Our findings describe temporal and regional variations in CRKP prevalence, AMR patterns, and genotypes, and offer new insights into the epidemiology, genomic diversity, and public health relevance of CRKP colonization in healthy individuals.

One notable observation in this study was a descriptive temporal change in carbapenemase gene composition, with *bla*_NDM_ variants, particularly *bla*_NDM‐5_. becoming more frequently detected in later years of the sampled cohort. The increased representation of *bla*_NDM‐5_ in later years may reflect a combination of lineage expansion, plasmid dissemination, and differences in sampling composition over time [12]. Although *bla*_NDM‐5_‐positive isolates were detected in multiple ST backgrounds, we did not perform a formal clone‐aware temporal analysis. Therefore, this pattern should be interpreted cautiously and not as a proven gene‐level replacement event. Notably, unlike previous clinical studies that primarily linked *bla*_NDM_ dissemination to IncX3 plasmids, we identified IncHI2‐IncHI2A plasmids as the predominant vectors for *bla*_NDM‐5_ in this cohort [13]. This association was supported by third‐generation Nanopore sequencing and conjugation experiments, which demonstrated the transferability of IncHI2‐IncHI2A plasmids carrying *bla*_NDM‐5_. These results highlight the need for continued monitoring of non‐IncX3 plasmid backbones involved in carbapenemase gene spread.

K1 and K2 are the most prevalent capsular types associated with hvKP in clinical settings, frequently implicated in community‐acquired infections such as liver abscesses, pneumonia, and sepsis [14, 15]. Their capsular polysaccharides (CPS) confer protection against host phagocytosis. In recent years, these hypervirulent strains have evolved into hv‐CRKP through the acquisition of carbapenemase‐encoding plasmids, such as those carrying *bla*_KPC_ or *bla*_NDM_, thereby significantly complicating clinical treatment [4]. Notably, in this study, only two K2‐type CRKP isolates were recovered from healthy individuals. Several factors may explain this observation. First, clinical isolates must evade host immune clearance and establish colonization in host tissues, and K1/K2 CPS contributes to this by enhancing resistance to phagocytosis and promoting biofilm formation, traits particularly advantageous in infection settings [16]. In contrast, CRKP colonizing healthy individuals typically exist as commensals and lack such selective pressures for virulence, leading to reduced prevalence of high‐virulence capsular types. Second, individuals with compromised immune function are more susceptible to infections by K1/K2‐type hvKP, which may partly explain their higher prevalence in clinical patients than in healthy populations.

In addition, we observed notable shifts in capsular types among CRKP isolates. Compared with clinical CRKP isolates, CRKP from healthy individuals exhibited fewer K types, primarily KL64 and KL47^6^. KL47 became more frequently observed than KL64 in later sampling years among healthy carriers, including among hv‐CRKP isolates. While KL64 is often associated with enhanced virulence and higher clinical mortality, the emergence of KL47 in community hv‐CRKP strains may reflect ecological adaptation to the gut environment, where virulence pressures are reduced, but persistence is favored [17]. These findings point to a potential reshaping of hypervirulent lineages outside clinical settings.

The ST11 lineage accounted for nearly half of all isolates and was detected repeatedly across multiple years, indicating its persistent presence in this cohort. Previous studies have shown that ST11 is a successful high‐risk clone with strong dissemination and persistence potential [18]. Consistent with these findings, our gene presence/absence association analysis identified enrichment in ST11 of loci related to capsular polysaccharide biosynthesis, iron acquisition, and secretion‐associated functions [19, 20, 21]. These functions are known to support host adaptation and fitness in *K. pneumoniae* and may be relevant to persistence in host‐associated environments [22]. However, because this analysis did not explicitly control for population structure or clonal relatedness, the identified loci should be interpreted as ST11‐associated candidate features rather than definitive determinants of asymptomatic intestinal persistence. These findings are therefore hypothesis‐generating and require further validation in lineage‐aware analyses and functional studies.

Finally, our comparison between ST11 isolates from healthy individuals and clinical patients revealed a high degree of genomic similarity, despite differences in ARG load. This finding indicates that healthy carriers may harbor CRKP strains closely related to clinical isolates. The close genomic relatedness observed here suggests potential epidemiological connections between colonizing and clinical CRKP populations, but it cannot determine transmission routes or directionality. Therefore, surveillance strategies that extend beyond symptomatic infections may provide a more complete picture of CRKP circulation.

This study has several limitations that warrant consideration. First, the samples were collected from individuals attending hospital‐based routine physical examinations, which may not be fully representative of the general community population and may introduce selection bias. Second, the stool detection workflow was based on routine laboratory procedures, and its sensitivity for detecting low‐abundance CRKP colonization was not formally assessed. Therefore, the prevalence estimates and regional/temporal trends reported here should be interpreted as reflecting the cohort derived from these participating hospitals rather than the broader community. Third, because this was a retrospective study and sampling intensity varied across provinces, hospitals, and years, we did not perform adjusted statistical modelling to estimate independent epidemiological effects. Fourth, the available temporal and epidemiological metadata were insufficient for robust time‐resolved phylogenetic reconstruction; therefore, transmission routes, evolutionary rates, and directionality could not be inferred. In addition, these upstream limitations may also affect downstream estimates of resistance profiles, carbapenemase/ARG distributions, and capsular or hypervirulence frequencies. These limitations mean that the observed geographic, temporal, and genomic patterns should be interpreted as descriptive associations and hypothesis‐generating findings rather than causal or mechanistic evidence.

## 4 Conclusion

This study provides the first longitudinal, nationwide genomic landscape of CRKP colonizing healthy individuals in China. We identified temporal variation in resistance determinants, plasmid backgrounds, and dominant clonal lineages within this sampled cohort, with ST11 remaining the predominant lineage and showing enrichment of candidate lineage‐associated loci. These findings suggest that healthy individuals in this hospital‐based cohort can harbor CRKP strains that are genomically related to clinically relevant isolates, supporting the value of surveillance beyond symptomatic infections.

## 5 Materials and Methods

### 5.1 Study Design and Sampling

Bacterial isolates from bowel samples were obtained from healthy individuals without evidence of intestinal infection from hospitals across 30 regions (Anhui, Beijing, Fujian, Gansu, Guangdong, Guangxi, Guizhou, Hainan, Hebei, Henan, Heilongjiang, Hubei, Hunan, Jilin, Jiangsu, Jiangxi, Liaoning, Inner Mongolia, Ningxia, Qinghai, Shandong, Shanxi, Shaanxi, Shanghai, Shenyang, Sichuan, Tibet, Xinjiang, Yunnan, Zhejiang provinces) in China in 2016 and from 2019 to 2024. These 16,040 nonduplicate stool samples were obtained from individuals who attended hospital‐based routine physical examinations. Therefore, the study population represents a health‐check cohort rather than a population‐based community sample [23]. Because this was a retrospective study, individual‐level information on prior antibiotic exposure and other potential risk factors was not uniformly available. All isolates were identified by matrix‐assisted laser desorption ionization‐time of flight mass spectrometry (bioMérieux, France). The presence of common carbapenem resistance genes (*bla*_IMP_, *bla*_NDM_, *bla*_VIM_, *bla*_KPC_, and *bla*_OXA‐48‐like_) was tested using the NG‐Test CARBA5 (NG Biotech, Guipry, France) [24].

### 5.2 Antimicrobial Susceptibility Testing (AST)

The determination of minimal inhibitory concentrations (MICs) was conducted in accordance with the reference broth microdilution technique endorsed by the Clinical and Laboratory Standards Institute (CLSI) [25]. The breakpoints of *K. pneumoniae* according to CLSI M100‐ED31 were interpreted for imipenem, meropenem, ertapenem, cefmetazole, ceftazidime, cefotaxime, piperacillin/tazobactam, cefoperazone/sulbactam, ceftazidime/avibactam, cefepime, ciprofloxacin, amikacin, and aztreonam. Tigecycline MICs were interpreted using the US Food and Drug Administration MIC breakpoints for Enterobacterales (https://www.fda.gov). European Committee on Antimicrobial Susceptibility Testing (EUCAST) MIC interpretive breakpoints were used for polymyxin B (Table 1; Table S1). *E. coli* ATCC 25922 and *K. pneumoniae* ATCC 700603 were used as quality control strains. *K. pneumoniae* strains showing resistance to at least one of the carbapenems, meropenem or imipenem, were designated as CRKP [26, 27].

### 5.3 Whole Genome Sequencing and De Novo Assembly

Whole genome sequencing was performed on 105 CRKP isolates using the Illumina NovaSeq 6000 platform. De novo genome assembly was performed using SPAdes version 3·15·1. According to the Illumina sequencing data, one representative isolate containing IncHI2‐IncHI2A plasmid replicons was selected for further sequencing by long‐read Nanopore sequencing. Complete genome sequences were obtained using Unicycler version 0.4.8 with the default parameters. The assembled genomes were annotated using PROKKA [28].

### 5.4 Screening ST11 CRKP and Hv‐CRKP Isolates in Retrospective Data

All *K. pneumoniae* genome sequences available at the National Center for Biotechnology Information (NCBI) genome database as of December 1, 2024 were retrieved. Basic information for each isolate, including country of origin, year of isolation, and accession number, was extracted using a custom Python script and compiled into an Excel spreadsheet. All ST11 CRKP carrying carbapenemase genes of patient origin were selected for further study. According to the definition proposed by Zhou et al. [6], isolates harboring *iuc* and at least one other virulence‐associated gene were classified as hvKP. The virulence‐associated genes considered in this study were *rmpA, rmpA2, iroB, iucA*, and *peg344*. Among these hvKP isolates, those additionally carrying carbapenem‐resistance genes (*bla*_IMP_, *bla*_NDM_, *bla*_VIM_, *bla*_KPC_, or *bla*_OXA‐48‐like_) were classified as hv‐CRKP.

### 5.5 Bioinformatics Analysis

Single‐nucleotide polymorphisms (SNPs) were identified using Parsnp v1.2. Isolates differing by ≤10 allelic differences were considered as a single clone [29]. Bioinformatic analyses, including species identity, multilocus sequence typing (MLST), and identification of ARGs of isolates, were performed using Kleborate version 2.2.0 [30]. Sequence types (STs) were determined by comparing the seven housekeeping genes in 105 strains. The seven conserved housekeeping genes were obtained from the public databases for molecular typing and microbial genome diversity (https://pubmlst.org). Plasmid replicons were identified using PlasmidFinder [31]. Virulence factors (*rmpA, rmpA2, iroB, iucA*, and *peg344*) were confirmed by the core dataset of the Virulence Factor Database (VFDB) [32]. Genomic islands were predicted using IslandViewer 4, and insertion sequences (ISs) were screened using ISfinder version 1.0 [33]. Kaptive software was also used to identify the capsule synthesis loci (K‐loci or KL) [34]. Phylogenetic trees of the isolates were constructed using Roary and FastTree based on SNPs in the core genomes [35, 36]. SNPs in the genomes of CRKP isolates were detected using snp‐dists version 0.7.0 (https://github.com/tseemann/snp‐dists). The trees were visualized and modified using iTOL version 6.5.7 [37]. The network graph describing the co‐occurrence of ARGs and ISs was constructed using Gephi (https://gephi.org).

To explore genomic differences associated with the predominant ST11 lineage, a gene presence/absence association analysis was performed using Scoary based on the pangenome‐derived gene presence/absence matrix [38]. Isolates were grouped into ST11 and non‐ST11 categories, and each gene was tested for differential distribution between the two groups. Statistical significance was determined using Bonferroni‐corrected *p‐*values, with genes below the predefined threshold (*p* < 0.05) considered significantly associated with the ST11 background. Because Scoary was applied to gene presence/absence data without explicit modeling of population structure or clonal relatedness, the resulting associations were interpreted as exploratory lineage‐associated signals.

### 5.6 Filter Mating Assay

To investigate the transferability of 3 type carbapenem‐resistance genes (*bla*_NDM_, *bla*_IMP_, and *bla*_KPC_), nine isolates were evaluated through conjugation experiments employing the filter mating assay [39]. The recipient strain used was hygromycin‐resistant *K. pneumoniae* YZ6. Transconjugants were selected on MH agar plates with 200 mg/L hygromycin and 2 mg/L meropenem or 8 mg/L ceftazidime‐avibactam. MALDI‐TOF/MS and PCR were used to confirm the presumptive transconjugants.

### 5.7 Statistical Analysis

Statistical analysis and plotting were performed using R version 4.2.2 (R Foundation for Statistical Computing, Vienna, Austria). Additionally, the Mantel test, Spearman correlation analysis, and Mann–Kendall trend test (MK test) were conducted to evaluate the relationships and temporal trends in resistance rates across different antibiotics, using R software for computation. *p* < 0.05 was considered statistically significant. Spearman correlation analysis was used to determine the correlation among ARGs, ISs, VFs, and plasmid replicons [40]. The association between ARGs and insertion sequences was analyzed based on the Spearman algorithm and visualized using Gephi (https://gephi.org). Statistical analyses were performed using either the chi‐squared (*χ* ^2^) test or Fisher's exact test via the IBM SPSS software (IBM, USA).

## Author Contributions

Ruichao Li and Rong Zhang conceived and supervised the study. Zelin Yan and Yan Li performed the experiments, collected the data, and prepared the draft. Zelin Yan, Qiaoling Sun, and Congcong Liu interpreted the data and conducted the analysis. Yanyan Zhang, Yuchen Wu, Hongwei Zhou, and Shangshang Qin contributed to sample collection and manuscript editing. Rong Zhang and Ruichao Li acquired the funding. All authors reviewed and approved the final manuscript.

## Funding Information

This work was supported by the National Key Research and Development Program of China (No. 2022YFD1800400) and the Theme‐based Research Scheme (T11‐104/22‐R), the China Postdoctoral Science Foundation (2025M771434), the Natural Science Foundation of Jiangsu Province (No. BK20231524), and the Priority Academic Program Development of Jiangsu Higher Education Institutions (PAPD).

## Ethics Statement

All of our research complies with the relevant ethical regulations. Ethical permission for this study was granted by the Ethics Committee of The Second Affiliated Hospital, Zhejiang University School of Medicine (2024‐1335). Verbal consent was obtained from the participants before their participation. All participants and data included in the study were anonymized [6].

## Conflicts of Interest

The authors declare no conflicts of interest.

## Supporting information




**Supporting Figure 1**: Circular comparison of *bla*_NDM‐5_‐positive IncHI2‐IncHI2A plasmid structures.
**Supporting Figure 2**: Distribution of virulence genes in CRKP isolates from healthy individuals in China.
**Supporting Figure 3**: Differences in capsular types between CRKP isolates from northern and southern China.
**Supporting Figure 4**: The correlation of counts of ARGs, plasmid replicons, insertion sequences, and VFs detected from genomes.
**Supporting Figure 5**: Distribution of CRKP STs and the ARGs and virulence genes carried by ST CRKP isolates.
**Supporting Figure S6**: The correlation of counts of ARGs, plasmid replicons, and insertion sequences detected from genomes of ST11 CRKP.
**Supporting Figure 7**: Phylogenetic analysis of ST11 CRKP from healthy individuals and clinical patients.
**Supporting Table 2**: Antimicrobial susceptibility profiles of recipient strain YZ6 and its transconjugants. SNPs among 105 CRKP isolates collected from healthy populations.
**Supporting Table 5**: Basic information of 113 CRKP collected from patients. Significantly different genes between ST11 and non‐ST11 CRKPs.

**Supporting Table 1**: Basic information of 105 CRKP isolates collected from healthy populations.

**Supporting Table 3**: SNPs among 105 CRKP isolates collected from healthy populations.

**Supporting Table 4**: Significantly different genes between ST11 and non‐ST11 CRKPs.

**Supporting Table 6**: SNPs among 161 CRKP isolates collected from healthy populations and patients.

## Data Availability

Genome sequence data reported in this study have been deposited in the National Center for Biotechnology Information under BioProject PRJNA1190336. All study data are included in the article and/or supporting information.
