# Supplementary material for: Longitudinal Molecular Epidemiology and Genomic Surveillance of Carbapenem‐Resistant Klebsiella pneumoniae in the Gut of Healthy Individuals in China
Source: MedComm (2020). 2026 Aug 5;7(8):e70814. doi: 10.1002/mco2.70814 (PMC13439402; doi:10.1002/mco2.70814)
Supplement: Supplementary file 1 [file MCO2-7-e70814-s004.docx]

**Supplementary Information**

**Longitudinal Molecular Epidemiology and Genomic Surveillance of Carbapenem-Resistant *Klebsiella pneumoniae* in the Gut of Healthy Individuals in China**

Zelin Yan^1#^, Yan Li^2,3#^, Qiaoling Sun^4^, Congcong Liu^1,5^, Yanyan Zhang^1^, Yuchen Wu^1^, Hongwei Zhou^1^, Shangshang Qin^2^, Ruichao Li^3*^, Rong Zhang^1*^

^1*^ Department of Clinical Laboratory, Second Affiliated Hospital of Zhejiang University, School of Medicine, Hangzhou, Zhejiang, China.

^2^ Key Laboratory of Advanced Drug Preparation Technologies, Ministry of Education, Zhengzhou University, Zhengzhou, Henan, China.

^3^ Jiangsu Co-Innovation Center for Prevention and Control of Important Animal Infectious Diseases and Zoonoses, College of Veterinary Medicine, Yangzhou University, Yangzhou, Jiangsu, China.

^4^ Department of Laboratory Medicine, the First Affiliated Hospital, Zhejiang University School of Medicine, Hangzhou, China.

^5^ Department of Clinical Laboratory, Tianjin First Central Hospital, School of Medicine, Nankai University, Tianjin, China.

^*^Corresponding authors: Ruichao Li, Email: [rchl88@yzu.edu.cn](mailto:rchl88@yzu.edu.cn)

Rong Zhang, Email: zhang-rong@zju.edu.cn

^#^These authors contributed equally to this work.

**Supplementary Figure and Table Legends**

**Figure S1. Circular comparison of *bla*_NDM-5_-positive IncHI2-IncHI2A plasmid structures.**

**Figure S2. Distribution of virulence genes in CRKP isolates from healthy individuals in China.**

**Figure S3.** **Differences in capsular types between CRKP isolates from northern and southern China.**

**Figure S4. The correlation of counts of ARGs, plasmid replicons, insertion sequences and VFs detected from genomes.**

**Figure S5. Distribution of CRKP STs and the ARGs and virulence genes carried by ST CRKP isolates.**

**Figure S6. The correlation of counts of ARGs, plasmid replicons and insertion sequences detected from genomes of ST11 CRKP.**

**Figure S7. Phylogenetic analysis of ST11 CRKP from healthy individuals and clinical patients.**

**Table S1. Basic information of 105 CRKP isolates collected from healthy populations.**

**Table S2. Antimicrobial susceptibility profiles of recipient strain YZ6 and its transconjugants.**

**Table S3. SNPs among 105 CRKP isolates collected from healthy populations.**

**Table S4. Significantly different genes between ST11 and non-ST11 CRKPs.**

**Table S5. Basic information of 113 CRKP collected from patients.**

**Table S6. SNPs among 161 CRKP isolates collected from healthy populations and patients.**

**This Supplementary Information file contains the legends for all supplementary figures and tables. Large supplementary tables (Table S1, Table S3, Table S4 and Table S6) are provided as separate files.**

**Figure S1. Circular comparison of *bla*_NDM-5_-positive IncHI2-IncHI2A plasmid structures.** The plasmid pH22-ZJ03-NDM-5 was used as a reference plasmid.

**Figure S2. Distribution of virulence genes in CRKP isolates from healthy individuals in China.** * Indicates *p* < 0.05, ** Indicates *p* < 0.01.

**Figure S3.** **Differences in capsular types between CRKP isolates from northern and southern China.** Purple represents capsular types unique to northern China, pink represents capsular types unique to southern China, and blue represents capsular types shared between both regions.

**Figure S4. The correlation of counts of ARGs, plasmid replicons, insertion sequences and VFs detected from genomes.** (A) The correlation between plasmid replicon and ARGs counts. (B) The correlation between insertion sequences and ARGs counts. (C) The correlation between ARGs and virulence gene counts.

**Figure S5. Distribution of CRKP STs and the ARGs and virulence genes carried by ST CRKP isolates.** (A) STs distribution of CRKP isolates in different years. (B) Temporal changes in ARGs carried by ST11 CRKP isolates. (C) Temporal changes in virulence genes carried by ST11 CRKP isolates.

**Figure S6. The correlation of counts of ARGs, plasmid replicons and insertion sequences detected from genomes of ST11 CRKP.** (A) Linear correlation between the number of ARGs and insertion sequences. (B) Linear correlation between the number of ARGs and plasmid replicons.

**Figure S7. Phylogenetic analysis of ST11 CRKP from healthy individuals and clinical patients.** Resistance genes are indicated by squares: solid square indicates has; hollow square indicates does not have.

| **Table S2. Antimicrobial susceptibility profiles of recipient strain YZ6 and its transconjugants.** | | | |  |
| --- | --- | --- | --- | --- |
| **Description** | **Recipent strains** | **Transconjugant of *bla*NDM -carrying plasmid** | |  |
| Strains | YZ6 | Y1 | Y2 |  |
| IMP | ≤ | 16 | 32 |  |
| MEM | ≤ | 32 | 128 |  |
| ETP | ≤ | 32 | 128 |  |
| CMZ | 4 | 8 | 64 |  |
| CAZ | 8 | >128 | >128 |  |
| CTX | >128 | >128 | >128 |  |
| TZP | >256/4 | >256/4 | >256/4 |  |
| SCF | 64/32 | >256/128 | >256/128 |  |
| CAV | ≤ | >64/4 | >64/4 |  |
| FEP | >64 | 32 | >64 |  |
| PB | ≤ | ≤0.5 | ≤0.5 |  |
| TGC | ≤ | 0.5 | ≤0.25 |  |
| CIP | 4 | ≤1 | 4 |  |
| AK | ≤4 | ≤4 | ≤4 |  |
| ATM | 64 | ≤4 | 16 |  |
| Abbreviations: IMP, imipenem; MEM, meropenem; ETP, ertapenem; CMZ, cefmetazole; CAZ, ceftazidime; CTX, cefotaxime; TZP, piperacillin/tazobactam; SCF, cefoperazone/sulbactam; CAV, ceftazidime/avibactam; FEP, cefepime; PB, polymyxin B; TGC, tigecycline; CIP, ciprofloxacin; AK, amikacin; ATM, aztreonam. | | | |  |
|  |  |  |  |  |

| **Table S5. Basic information of 113 CRKP collected from patients.** | | | | |  |
| --- | --- | --- | --- | --- | --- |
| ID | Biosample | Year | Source | Country | |
| GCA_022517925.1 | SAMN25988412 | 2016 | patient | United Kingdom | |
| GCA_022528465.1 | SAMN25989525 | 2016 | patient | United Kingdom | |
| GCA_022529745.1 | SAMN25988871 | 2016 | patient | United Kingdom | |
| GCA_022531025.1 | SAMN25989695 | 2016 | patient | United Kingdom | |
| GCA_022535735.1 | SAMN25989649 | 2016 | patient | United Kingdom | |
| GCA_022532465.1 | SAMN25989145 | 2016 | patient | United Kingdom | |
| GCA_022052845.1 | SAMN12886353 | 2019 | patient | China | |
| GCA_022052865.1 | SAMN12886354 | 2019 | patient | China | |
| GCA_022052885.1 | SAMN12886355 | 2019 | patient | China | |
| GCA_022052965.1 | SAMN12886356 | 2019 | patient | China | |
| GCA_022053065.1 | SAMN12886360 | 2019 | patient | China | |
| GCA_022053085.1 | SAMN12886362 | 2019 | patient | China | |
| GCA_022053185.1 | SAMN12886365 | 2019 | patient | China | |
| GCA_022053285.1 | SAMN12886346 | 2019 | patient | China | |
| GCA_022053685.1 | SAMN12886350 | 2019 | patient | China | |
| GCA_022053705.1 | SAMN12886349 | 2019 | patient | China | |
| GCA_022116735.1 | SAMN12886345 | 2019 | patient | China | |
| GCA_022116795.1 | SAMN12886358 | 2019 | patient | China | |
| GCA_022052805.1 | SAMN12886344 | 2019 | patient | China | |
| GCA_022052905.1 | SAMN12886357 | 2019 | patient | China | |
| GCA_022052945.1 | SAMN12886343 | 2019 | patient | China | |
| GCA_022053025.1 | SAMN12886361 | 2019 | patient | China | |
| GCA_022053045.1 | SAMN12886359 | 2019 | patient | China | |
| GCA_022053105.1 | SAMN12886363 | 2019 | patient | China | |
| GCA_022053125.1 | SAMN12886366 | 2019 | patient | China | |
| GCA_022053165.1 | SAMN12886364 | 2019 | patient | China | |
| GCA_022053505.1 | SAMN12886347 | 2019 | patient | China | |
| GCA_022053665.1 | SAMN12886348 | 2019 | patient | China | |
| GCA_022053725.1 | SAMN12886351 | 2019 | patient | China | |
| GCA_022053745.1 | SAMN12886352 | 2019 | patient | China | |
| GCA_031011165.1 | SAMN26993969 | 2018 | patient | China | |
| GCA_031011185.1 | SAMN26993970 | 2018 | patient | China | |
| GCA_031011585.1 | SAMN26993971 | 2018 | patient | China | |
| GCA_031011755.1 | SAMN26993972 | 2018 | patient | China | |
| GCA_031013065.1 | SAMN26993978 | 2019 | patient | China | |
| GCA_031011985.1 | SAMN26993973 | 2019 | patient | China | |
| GCA_031012205.1 | SAMN26993974 | 2019 | patient | China | |
| GCA_027942215.1 | SAMN27398041 | 2019 | patient | China | |
| GCA_031012425.1 | SAMN26993975 | 2019 | patient | China | |
| GCA_031012665.1 | SAMN26993976 | 2019 | patient | China | |
| GCA_031012885.1 | SAMN26993977 | 2019 | patient | China | |
| GCA_031011705.1 | SAMN26993993 | 2019 | patient | China | |
| GCA_031011645.1 | SAMN26993992 | 2019 | patient | China | |
| GCA_031011725.1 | SAMN26993996 | 2019 | patient | China | |
| GCA_031011685.1 | SAMN26993998 | 2019 | patient | China | |
| GCA_031011745.1 | SAMN26993999 | 2019 | patient | China | |
| GCA_027942195.1 | SAMN27398042 | 2019 | patient | China | |
| GCA_031011805.1 | SAMN26994001 | 2019 | patient | China | |
| GCA_031011845.1 | SAMN26994004 | 2019 | patient | China | |
| GCA_031011885.1 | SAMN26994003 | 2019 | patient | China | |
| GCA_031011925.1 | SAMN26994007 | 2019 | patient | China | |
| GCA_031011405.1 | SAMN26993980 | 2019 | patient | China | |
| GCA_031011515.1 | SAMN26993985 | 2019 | patient | China | |
| GCA_031011565.1 | SAMN26993986 | 2019 | patient | China | |
| GCA_031011485.1 | SAMN26993987 | 2019 | patient | China | |
| GCA_031011625.1 | SAMN26993989 | 2019 | patient | China | |
| GCA_031011665.1 | SAMN26993991 | 2019 | patient | China | |
| GCA_031012085.1 | SAMN26994013 | 2020 | patient | China | |
| GCA_031012065.1 | SAMN26994014 | 2020 | patient | China | |
| GCA_031012185.1 | SAMN26994016 | 2020 | patient | China | |
| GCA_031012025.1 | SAMN26994011 | 2020 | patient | China | |
| GCA_031012045.1 | SAMN26994012 | 2020 | patient | China | |
| GCA_031012165.1 | SAMN26994017 | 2020 | patient | China | |
| GCA_031012125.1 | SAMN26994018 | 2020 | patient | China | |
| GCA_031012245.1 | SAMN26994019 | 2020 | patient | China | |
| GCA_031012265.1 | SAMN26994020 | 2020 | patient | China | |
| GCA_031012305.1 | SAMN26994022 | 2020 | patient | China | |
| GCA_027942175.1 | SAMN27398043 | 2020 | patient | China | |
| GCA_031012385.1 | SAMN26994028 | 2020 | patient | China | |
| GCA_031012485.1 | SAMN26994031 | 2020 | patient | China | |
| GCA_031012345.1 | SAMN26994024 | 2020 | patient | China | |
| GCA_031012365.1 | SAMN26994026 | 2020 | patient | China | |
| GCA_031012445.1 | SAMN26994029 | 2020 | patient | China | |
| GCA_031012465.1 | SAMN26994030 | 2020 | patient | China | |
| GCA_031012615.1 | SAMN26994037 | 2020 | patient | China | |
| GCA_031012685.1 | SAMN26994039 | 2020 | patient | China | |
| GCA_031012645.1 | SAMN26994038 | 2020 | patient | China | |
| GCA_031013045.1 | SAMN26994056 | 2020 | patient | China | |
| GCA_031012535.1 | SAMN26994033 | 2020 | patient | China | |
| GCA_031012765.1 | SAMN26994043 | 2020 | patient | China | |
| GCA_031012785.1 | SAMN26994044 | 2020 | patient | China | |
| GCA_031012825.1 | SAMN26994046 | 2020 | patient | China | |
| GCA_031012745.1 | SAMN26994042 | 2020 | patient | China | |
| GCA_031012845.1 | SAMN26994047 | 2020 | patient | China | |
| GCA_031012865.1 | SAMN26994048 | 2020 | patient | China | |
| GCA_031012905.1 | SAMN26994049 | 2020 | patient | China | |
| GCA_031012945.1 | SAMN26994053 | 2020 | patient | China | |
| GCA_031013005.1 | SAMN26994054 | 2020 | patient | China | |
| GCA_031012805.1 | SAMN26994045 | 2020 | patient | China | |
| GCA_031013025.1 | SAMN26994055 | 2020 | patient | China | |
| GCA_031013125.1 | SAMN26994061 | 2020 | patient | China | |
| GCA_031013085.1 | SAMN26994057 | 2020 | patient | China | |
| GCA_031013305.1 | SAMN26994058 | 2020 | patient | China | |
| GCA_031013105.1 | SAMN26994059 | 2020 | patient | China | |
| GCA_031013145.1 | SAMN26994060 | 2020 | patient | China | |
| GCA_031013165.1 | SAMN26994062 | 2020 | patient | China | |
| GCA_031012975.1 | SAMN26994051 | 2020 | patient | China | |
| GCA_031011205.1 | SAMN26994069 | 2020 | patient | China | |
| GCA_031011255.1 | SAMN26994070 | 2020 | patient | China | |
| GCA_031011245.1 | SAMN26994071 | 2020 | patient | China | |
| GCA_031013185.1 | SAMN26994063 | 2020 | patient | China | |
| GCA_031013205.1 | SAMN26994064 | 2020 | patient | China | |
| GCA_031013225.1 | SAMN26994065 | 2020 | patient | China | |
| GCA_031013245.1 | SAMN26994066 | 2020 | patient | China | |
| GCA_031013325.1 | SAMN26994068 | 2020 | patient | China | |
| GCA_031011225.1 | SAMN26994072 | 2020 | patient | China | |
| GCA_031011345.1 | SAMN26994076 | 2020 | patient | China | |
| GCA_031013265.1 | SAMN26994067 | 2020 | patient | China | |
| GCA_031011365.1 | SAMN26994078 | 2020 | patient | China | |
| GCA_031011385.1 | SAMN26994077 | 2020 | patient | China | |
| GCA_031011305.1 | SAMN26994073 | 2020 | patient | China | |
| GCA_031011285.1 | SAMN26994074 | 2020 | patient | China | |
| GCA_031011325.1 | SAMN26994075 | 2020 | patient | China | |
|  |  |  |  |  | |
